# A framework for the organization and delivery of systemic treatment

**DOI:** 10.3747/co.v16i1.297

**Published:** 2009-01

**Authors:** T. Vandenberg, N. Coakley, J. Nayler, C. DeGrasse, E. Green, J.A. Mackay, C. McLennan, A. Smith, L. Wilcock, M.E. Trudeau

**Affiliations:** * London Health Sciences Centre, London, ON; † Cancer Care Ontario’s Program in Evidence-Based Care, Department of Clinical Epidemiology and Biostatistics, McMaster University, Hamilton, ON; ‡ Lakeridge Health, Oshawa, ON; § The Ottawa Hospital, Ottawa, ON; || Cancer Care Ontario, Toronto, ON; # Cancer Centre of Southeastern Ontario, Kingston, ON; ** Odette Cancer Centre, Toronto, ON

**Keywords:** Chemotherapy, organizational policy, health care facilities, health care policy, systemic treatment

## Abstract

**Background:**

Increasing systemic treatment and shortages of oncology professionals in Canada require innovative approaches to the safe and effective delivery of intravenous (IV) cancer treatment. We conducted a systematic review of the clinical and scientific literature, and an environmental scan of models in Canada, the United Kingdom, Australia, and New Zealand. We then developed a framework for the organization and delivery of IV systemic treatment.

**Methods:**

The systematic review covered the medline, embase, cinahl, and HealthStar databases. The environmental scan retrieved published and unpublished sources, coupled with a free key word search using the Google search engine. The Systemic Treatment Working Group reviewed the evidence and developed a draft framework using evidence-based analysis, existing recommendations from various jurisdictions, and expert opinion based on experience and consensus. The draft was assessed by Ontario stakeholders and reviewed and approved by Cancer Care Ontario.

**Results:**

The poor quantity and quality of the evidence necessitated a consensus-derived model. That model comprises four levels of care determined by a regional systemic treatment program and three integrated structures (integrated cancer programs, affiliate institutions, and satellite institutions), each with a defined scope of practice and a specific organizational framework.

**Interpretation:**

New models of care are urgently required beyond large centres, particularly in geographically remote or rural areas. Despite limited applicable evidence, the development and successful implementation of this framework is intended to create sustainable, accessible, quality care and to measurably improve patient outcomes.

## 1. INTRODUCTION

In Canada, cancer is a major cause of morbidity and mortality, and the leading cause of potential years of life lost. Increasing demands for cancer services relate directly to annual cancer incidence increases of 3%, resulting mainly from population growth and aging 1. Medical oncology consultations are increasing by 10%–20% annually, and systemic treatment has increased at an annual rate of 7%–10%, fuelled by new evidence-based therapies that improve survival and quality of life 2. Even more enhanced treatments are predicted 3. These treatments are often more complex than those they replace, and they are delivered for longer periods as the survival time with cancer—now increasingly a chronic disease—lengthens.

Several provincial and national bodies have convincingly demonstrated that ongoing clinical human resource shortages limit funding and filling of new oncology positions 2,4–9. For example, the November 2005 Canadian Post-md Education Registry revealed that only 34 medical oncology residents and 8 fellows are in oncology training for the entire country 5. Exacerbating this problem are the numbers of retiring physicians 6 and registered nurses 8 anticipated in Canada.

Given such changes, Canadian provinces need to devise innovative ways to deliver safe and effective systemic treatment in an ambulatory care setting for people with cancer. The risks of not pursuing a revised and sustainable model of systemic treatment delivery include adoption of ad hoc and inconsistent local solutions; cessation of service in some jurisdictions; and inequalities in access to, and standards of, care.

The purpose of the present work was to provide a practical framework to guide standardized delivery of evidence-based systemic treatment in hospitals outside regional cancer centres, with special consideration for geographically dispersed regions. The primary goal of the framework is to provide safe, evidence-based systemic cancer treatment while maximizing the efficient use of resources and implementing the principle of patient-centred care provided as close to home as possible. Service provision; complexity of care; safety, accessibility, and quality across all care levels defined from the patient, organization, and system perspective; and appropriateness, transparency, and accountability were all taken into consideration. Quality, research, and education are interlinked and integral parts in the regional delivery of safe systemic treatment, ensuring the dissemination of new or improved evidence-based standards in this rapidly changing field.

## 2. METHODS

### 2.1 Evidence Base

We used two core methodologies to develop this framework:

- a systematic review of scientific and clinical research evidence and
- an environmental scan of systemic treatment models developed in other jurisdictions.

The scientific and clinical literature was systematically searched for published and unpublished reports pertaining to the organization and delivery of systemic treatment. Published sources included the medical databases medline (ovid, 1996 through June 2006), embase (ovid, 1996 through June 2006), cinahl (ovid, 1996 through June 2006), and HealthStar (ovid, 1996 through June 2006). The terms used were “anti-neoplastic agents,” “chemotherapy,” “infusions intravenous,” and “neoplasms,” combined with “health facilities,” “organizational policy,” “continuity of patient care,” “outpatient clinics,” “ambulatory care facilities,” “hospitals rural,” “hospitals community,” “hospitals general,” “health care facilities,” and “health care policy”. Article bibliographies and personal files were also searched for relevant evidence.

The environmental scan retrieved published and unpublished sources (June 25–July 4, 2006) documenting systemic treatment delivery at hospitals outside a larger cancer centre. Documents from countries with health care systems similar to Canada’s (United Kingdom, Australia, and New Zealand) were considered. In addition, a free keyword search was conducted through the Internet Google search engine, and a search was made for documents mentioned in the text or references of identified reports.

The inclusion criteria were kept purposefully broad. Any study design was considered if it provided evidence on ways to deliver systemic treatment within ambulatory care institutions. Outcomes of interest included health care provider roles and education, service type and complexity, service volumes, quality assurance, facility requirements, and administrative and organizational responsibilities. Specific details of the development of the evidence base can be found online at www.cancercare.on.ca/pdf/pebc12-10s.pdf 10.

### 2.2 Development of the Framework

The Regional Models of Care Systemic Treatment Working Group, comprising medical oncologists, a Cancer Care Ontario regional vice president, a regional cancer program administrator, a systemic treatment satellite nursing administrator, oncology nurses, administrators, pharmacists, and other professionals, reviewed the evidence and used a combination of evidence-based analysis, existing recommendations from various jurisdictions, and expert opinion based on experience and consensus to develop the framework. Given that the quantity and quality of evidence was generally poor, the panel agreed upon the framework elements through consensus of expert opinion.

A draft framework document was circulated to 191 stakeholders in Ontario for feedback, with 89% of respondents endorsing the framework and document recommendations. More details regarding the feedback obtained can be found at www.cancercare.on.ca/pdf/pebc12-10s.pdf 10. The document was also reviewed and approved by Cancer Care Ontario’s Clinical Council, Provincial Leadership Council, and Executive Team.

## 3. RESULTS

### 3.1 Evidence Base

Evidence on the current organization and delivery of systemic treatment across Canada, the United Kingdom, Australia, and New Zealand was gathered through a systematic search of the literature and a scan of documents from organizations concerned with systemic treatment quality practice. In Canada, the provinces of British Columbia, Saskatchewan, and Nova Scotia have instituted important initiatives^11^–^13^. More details of the evidentiary base considered by the panel can be found at www.cancercare.on.ca/pdf/pebc12-10s.pdf^10^.

### 3.2 Regional Models of Care for Systemic Treatment

**FIGURE 1:**
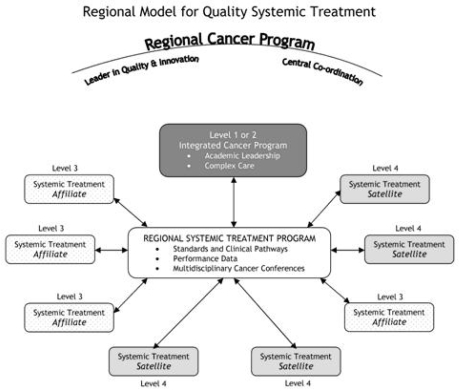
Regional model for quality systemic treatment. (Adapted from the Champlain Regional Cancer Surgery Model, 2006^14^)

The Regional Model for Quality Systemic Treatment (FIGURE 1) consists of a key set of fundamental elements and regional programs designed to implement, monitor, and evaluate quality indicators related to the delivery of safe, evidence-based, and patient-centred care. The model is an organizational framework for the delivery of systemic treatment within a regional systemic therapy program (rstp). The main goal of the model is to facilitate the provision of appropriate care in the appropriate setting within the appropriate time frame for all patients, regardless of the geographic location in which a patient receives systemic treatment. The model is composed of three integrated structures—integrated cancer programs (icps), affiliate institutions, and satellite institutions—each with a defined scope of practice.

The icps are multidisciplinary organizations that provide complex cancer care and that conduct cancer site–specific multidisciplinary care conferences (mccs). The mccs discuss unusual cases, oversee quality assurance, and provide assistance on cases seen at the rstp level 1–4 facilities in their own icp region. The mccs include surgical, radiation, and systemic therapy oncologists, nurses, pharmacists, social workers, pathologists, and radiologists. The icps provide leadership in the development of local guidelines for their region; they collect and assist in the analysis of outcome measures and quality indicators for funding, patient safety, and program organization and efficiency; and they may provide academic leadership, including educational support and access to research.

Affiliate institutions have their own systemic treatment programs, although they are linked through formal agreements with the rstp. Satellite institutions have fewer oncology-related resources and have a formal linkage to the rstp for support in delivering systemic treatment.

All regional partner institutions will participate in the development of their rstps and will collaboratively determine the appropriate configuration for their model, including the formal linkages that will be required among institutions. The complexity of care delivered in each type of institution may vary; standards encompassing four institutional levels of care (levels 1–4) are recommended for the delivery of systemic treatment, with the level of complexity and the availability of services differentiating one level from another. The rstp determines the appropriate level of care for each institution. Levels are hierarchical, with the satellite responsibilities being encompassed within the affiliate and icp levels. The designation of a level requires that an institution meet all the standards for that level. As individual institutions expand or focus their services, the configuration of the model and the designation of institutional levels may change, after consultation between the rstp and the institution.

The successful implementation of the framework is intended to create sustainable, accessible quality care and to measurably improve patient outcomes. The four levels and their standards are these:

- Level 4 (Satellite)
  - Ambulatory facilities, nursing, pharmacy, and physician support provided for the administration of low-risk to high-risk intravenous systemic treatment under the direction of an oncologist from an icp or an affiliate level 3 institution
  - Systemic treatments given under the supervision of a physician with appropriate oncology training
  - Access to specialized services and to providers with a formalized linkage to the rstp are ensured
- Level 3 (Affiliate)
  - Systemic treatments given under direct supervision of an on-site staff medical oncologist, hematologist, or gynecologic oncologist
  - Limited teaching and research responsibilities
- Level 2 (icp)
  - Systemic treatments given in a setting providing radiation treatment services and capable of providing most complex systemic treatments, including concurrent head-and-neck chemoradiation or radiolabelled conjugates (or both)
  - Limited teaching and research responsibilities
- Level 1 (icp)
  - Academic institutions with teaching and research responsibilities
  - Experimental investigational new drug (ind) program (ind phase i and ii trials) with highly developed clinical trials infrastructure—for example, participation in the National Cancer Institute of Canada Clinical Trials Group ind program and Princess Margaret Hospital/U.S. National Institutes of Health phase II new drug consortium
  - Responsible for training future health care professionals, including oncologists (subspecialty residents and fellows)

### 3.3 Defining Features for Each Level in the Framework

The goal of the rstp is to ensure safe, standardized, evidence-based care across the regions and equitable access to systemic treatment. TABLES I–VIII delineate the defining features by level in the areas of type of care, health care providers, education, service type and complexity, volumes, quality assurance and safety, facility requirements, and administrative responsibilities. Definitions for key terms are provided in Appendix a.

## 4. DISCUSSION AND CONSENSUS

The Regional Models of Care Systemic Treatment Project Team used the modest evidence that was available from the published literature and the environmental scan and the expert opinion of the membership to reach consensus on the defining features for the organization and for delivery of systemic cancer treatment.

For several years, Cancer Care Ontario regional networks delivered systemic treatment, particularly in rural areas, under a hub-and-spoke model; however, this delivery was accomplished without regional governance or management authority^17^. The provincial standard of care now is the new integrated regional systemic treatment model, with all its defining elements (FIGURE 1), which is being implemented with the goal of improving equitable access to appropriate evidence-based and coordinated cancer services. Existing regional cancer programs and new ones currently being developed in Ontario will be expected to meet the model requirements.

Although developed in the Ontario context, this model may, we believe, be useful in other provinces. The goal of the model is to ensure that, regardless of where in a province a patient receives systemic treatment, the same standard of care is guaranteed: the patient receives appropriate care in the appropriate setting within the appropriate time frame by clinicians with the expertise to offer the services. A regional program model replaces a traditional hub-and-spoke model and better reflects the relationships between all partners delivering systemic treatment.

In the new model, the rstp assumes regional leadership for the delivery of systemic treatment, with support from the provincial cancer organization. Although most regional authorities in Canada (for example, local health integration networks, district health councils, health authorities, or health regions) have icps, it is important to acknowledge that, to best meet patient needs, cross-regional collaboration must also be considered in the planning of rstps. In addition, regional authorities without icps also exist, and therefore regional cancer services must be planned through a neighbouring icp. Under the rstp, systemic treatment icps, affiliates, and satellites would work collaboratively to ensure safe, evidence-based care that maximizes the capacity of care given across the region, while ensuring appropriate high-quality care.

## 5. CONCLUSIONS

The structure for systemic treatment delivery in ambulatory centres provides a comprehensive regional and provincial framework. This framework has been formed through a combination of evidence and expert consensus. Consensus was achieved through a small working group and the larger Regional Models of Care Systemic Treatment Project Team. The framework outlines the four levels (institution types) of care that are recommended for the delivery of systemic treatment. A hospital is not prevented from moving up to the next level, provided that all the model requirements are met and that the rstp agrees to the move. The present work provides a framework for all hospitals to meet the same standards and, at the same time, to achieve quality care and service when administering systemic treatment. Although the present article has been created to sustain the Ontario Cancer Care network in providing safe and accessible care, we believe that it is applicable to, and useful for, other jurisdictions.

## 7. CONFLICT OF INTEREST NOTIFICATION

All authors declared no conflicts of interest.

## Figures and Tables

**TABLE I:** Type of systemic treatment care

Complexity	Level 1 (ICP)	Level 2 (ICP)	Level 3 (Affiliate)	Level 4 (Satellite)
Experimental investigational new drug program	Yes	No	No	No
Concurrent head-and-neck chemorads, or radiolabelled conjugates, or both	Yes	Yes	No	No
Oncologist on site determines treatment plan	Yes	Yes	Yes	No
All other systemic treatment	Yes	Yes	Yes	Yes

icp = integrated cancer program; chemorads = chemotherapy plus radiation therapy.

**TABLE II:** Health care providers and their roles

All levels	Where the standard identifies that services are to be provided in a multidisciplinary environment, all providers required for the service at a particular level are available or readily accessible.		
	All patients being considered for systemic treatment must be assessed by an oncologist.		
	All treatment plans are recommended by and parenteral systemic treatment is prescribed by the consulting oncologist.		
	Individual treatments as part of an approved course may be ordered by a family physician or internist with oncology training.		
	Ongoing care must be coordinated with the consulting oncologist.		
	Only registered nurses with appropriate chemotherapy certification may administer parenteral drugs.		
	Only pharmacists or pharmacy technicians will prepare systemic treatment.		
Oncologists	Level 4	(Satellite)	Access to oncologist from a level 1, 2, or 3 hospital is required to determine and recommend the treatment plan, to manage disease status, and to discuss patient management issues with the health care team.
	Level 3	(Affiliate)	One or more oncologists are on staff and on site.
			Mentor family physicians/internists.
			Provide limited teaching and research.
	Level 2	(icp)	Level 3, plus:
			Developed specific subspecialized practices.
	Level 1	(icp)	Level 2, plus:
			Have academic responsibilities, including teaching and research.
Family physicians/internists	Level 4	(Satellite)	Supervise intravenous systemic treatment administration with one physician on site or readily available (within 15 minutes) during the drug administration time.
			Consult oncologists regarding patient management issues (for example, dose alteration).
			Assess and manage toxicity.
			Participate in education programs related to the management of patients receiving systemic treatment.
	Level 3	(Affiliate)	Same as level 4.
	Level 2	(icp)	Same as level 3.
	Level 1	(icp)	Same as level 2.
Nurses	Level 4	(Satellite)	Administer systemic treatment, including monitoring and intervening for side effects and reactions, and provide supportive care to the patient.
			Provide patient education related to planned systemic treatment in collaboration with pharmacist and physicians.
			Communicate with icp or affiliate team members and collaborate with supervising physicians as necessary.
			Manage symptoms.
	Level 3	(Affiliate)	Level 4 plus:
			Plans to implement advanced oncology nurse.
	Level 2	(icp)	Level 3 plus:
			Specialized oncology nurses working towards the national certification—Certified in Oncology Nursing (Canada)—within 5 years of employment.
			Advanced oncology nurse to manage selected patient populations independently or interdependently with oncologists.
	Level 1	(icp)	Same as level 2.
Pharmacists	Level 4	(Satellite)	Review and verify systemic treatment orders and supervise the preparation and dispensing of systemic treatment.
			Consult with icp or affiliate pharmacist as required.
			Manage the reimbursement process for new drug funding program (pharmacist or pharmacy technician).
			Provide patient education related to medications in collaboration with nurses and physicians.
			Supervise and manage dispensing and documentation of clinical trials.
	Level 3	(Affiliate)	Same as level 4
	Level 2	(icp)	Level 3, plus:
			Provide support and consultation to regional systemic treatment program.
			Provide clinical services (dedicated oncology pharmacists).
	Level 1	(icp)	Same as level 2.
Pharmacy technicians	Level 4	(Satellite)	Prepare systemic treatment under supervision of a pharmacist.
	Level 3	(Affiliate)	Same as level 4.
	Level 2	(icp)	Same as level 3.
	Level 1	(icp)	Same as level 2.

icp = integrated cancer program.

**TABLE III:** Education of health care providers

All levels of care	Minimum standards are met for orientation and for annual continuing education and mentoring in systemic treatment for all staff working in oncology services.		
	Providers are competent to provide the designated level of service and have ongoing education to maintain that competence.		
	Registered nurses meet organizational policy and standards to be certified in chemotherapy administration.		
Oncologists	Level 4	(Satellite)	—
	Level 3	(Affiliate)	Take ongoing continuing medical education per the Royal College of Physicians and Surgeons of Canada.
			Participate in multidisciplinary cancer conferences as required.
	Level 2	(icp)	Same as level 3.
	Level 1	(icp)	Same as level 2.
Family physicians/internists	Level 4	(Satellite)	Take initial orientation and annual continuing medical education.
			Mentoring by an oncologist should be available.
			Relevant training for systemic treatment being delivered.
			Know the systemic treatment guidelines and standards, and regional policies and procedures.
			Participate in multidisciplinary cancer conferences as required.
	Level 3	(Affiliate)	Same as level 4.
	Level 2	(icp)	Same as level 3.
	Level 1	(icp)	Same as level 2
Nurses	Level 4	(Satellite)	Registered nurse certified for the delivery of systemic treatment.
			Working towards Certified in Oncology Nursing (Canada) or recertification, or both.
			Educated in central venous access device management and selection, certification. Annual update required.
			Oriented to and practicing according to the
			safe handling of cytotoxic agents standards, and
			central venous access devices guidelines.
			Participation in multidisciplinary cancer conferences encouraged.
			Additional ongoing education required to match treatment type and complexity.
	Level 3	(Affiliate)	Level 4, plus:
			Registered nurse specialized in oncology, certified in systemic treatment administration, and annually updated in guidelines and procedures.
			Plans to implement advanced oncology nursing roles.
	Level 2	(icp)	Level 3, plus:
			Specialized oncology nurses working towards Certified in Oncology Nursing (Canada); certification should be obtained within 5 years of new employment.
			Advanced oncology nurse (clinical nurse specialist or acute care nurse practitioner, Master’s preparation) with additional knowledge and skills in managing patients on systemic treatment.
			Additional education for nurses managing transplant patients.
	Level 1	(icp)	Same as level 2.
Pharmacists	Level 4	(Satellite)	Specialized training in oncology.
			Regional systemic treatment program should provide a training or certification program for staff involved in the handling of cytotoxic agents and should have a policy on retraining. Programs may be held at or in collaboration with an icp or affiliate institution.
			Training may include institutional training or orientation program for oncology pharmacists, continuing education programs or courses, oncology pharmacy review courses (for example, American Society of Health-System Pharmacists oncology review course), preceptorship programs.
	Level 3	(Affiliate)	Same as level 4.
	Level 2	(icp)	Same as level 3.
	Level 1	(icp)	Same as level 2.
Pharmacy technicians	Level 4	(Satellite)	Specialized training in the preparation of systemic treatment doses.
			Regional systemic treatment program should provide a training or certification program for staff involved in the handling of cytotoxic agents and have a policy on retraining. Programs may held at or in collaboration with an icp or affiliate institution.
be			
	Level 3	(Affiliate)	Same as level 4.
	Level 2	(icp)	Same as level 3.
	Level 1	(icp)	Same as level 2.

icp = integrated cancer program.

**TABLE IV:** Service type and complexity

All levels of care	Services are provided in the most appropriate setting, where patients can be assured the best-quality outcomes.		
	Each level has access to the other levels where necessary, for consultation or for transfer for service delivery.		
Service type	See the disease-site-specific Cancer Care Ontario core and core restricted regimens as an example: www.cancercare.on.ca/index_chemoRegimensbyDisease.htm.		
	Clinical trial drugs will be given at level 1, 2, or 3, or at level 4 under the supervision of oncologist.		
	Levels 4 and 3	(Satellite, Affiliate)	Per the example of Cancer Care Ontario core and core restricted regimens.
	Level 2	(icp)	Per the example of core and core restricted regimens, plus:
			Concurrent chemotherapy and radiation.
			Radiopharmaceuticals.
	Level 1	(icp)	Same as level 2.
Complexity	Level 4	(Satellite)	Low to high complexity.
			Assessment for and management and coordination of central venous access devices (such as a peripherally inserted central catheter or Port-A-Cath).
			Drugs with a high risk of hypersensitivity reaction at first dose will be given only at level 4 centres as agreed upon by the regional systemic treatment program.
			Delivery of systemic treatment in presence of comorbidity or significant organ dysfunction that increases risk of toxicity and need for dose adjustments, if agreed upon by regional systemic treatment program.
			Monitoring and management of hypersensitivity reactions.
	Level 3	(Affiliate)	Same as level 4, plus:
			Delivery of first-dose high-risk drugs.
			High complexity.
	Level 2	(icp)	Level 3, plus:
			Provision of on-site direct coordination and supervision of medical and radiation treatment.
			Pathology consultation on site.
	Level 1	(icp)	Same as level 2
Patient education	Level 4	(Satellite)	If possible, on-site patient education program that meets the Cancer Care Ontario standards.
	Level 3	(Affiliate)	Same as level 4, plus:
			Adhere to Cancer Care Ontario patient education standards.
	Level 2	(icp)	Level 3, plus:
			Patient education program related to radiation treatment.
	Level 1	(icp)	Level 2, plus:
			Patient education related to investigational treatments
Supportive care	Level 4	(Satellite)	Access to supportive care services to address specific patient needs.
	Level 3	(Affiliate)	Same as level 4.
	Level 2	(icp)	Comprehensive supportive care expertise as part of icp.
	Level 1	(icp)	Same as level 2.
Clinical trials	Level 4	(icp)	If clinical trials are conducted at the institution, they must be under the direction of an oncologist.
			Family physicians or internists with oncology training may be co-investigators.
			Specific clinical trial education for patients and health care providers.
	Level 3	(Affiliate)	Specific clinical trial education for patients and health care providers.
			Clinical trials including phases ii and iii.
	Level 2	(icp)	Same as level 3
	Level 1	(icp)	Same as level 2, plus:
			Investigational new drug program with phase i or ii drugs, or both.

icp = integrated cancer program.

**TABLE V:** Service volumes

All levels of care	The location has sufficient patient volume to maintain competency and skills of professional providers to address the acuity and complexity of the treatment modalities and to provide cost-effective use of resources and drugs.
	The number of patients that can be treated will be affected by the complexity of the treatment regimens.
	Staffing must be sufficient to provide safe, quality care at all times, including during vacation, illness, and so on.

**TABLE VI:** Quality assurance and safety

All levels of care	Cancer care includes management of complications of therapy.		
	All centres will follow the safe handling of cytotoxic agents standards.		
	Up-to-date guidelines from the regional systemic treatment program are available for staff for relevant disease sites and relevant symptom management.		
	Training and guidelines include management of oncology emergencies.		
	Access to specialized centres (icp level 1 or 2 or affiliate level 3) for support of quality and standards.		
	Provision of systemic treatment in the most effective manner.		
Safe handling	Level 4	(Satellite)	Policies and education programs available for all staff involved in systemic treatment, including storage, transport, spill management, preparation, administration, and waste disposal.
	Level 3	(Affiliate)	Same as level 4.
	Level 2	(icp)	Same as level 3.
	Level 1	(icp)	Same as level 2.
Patient outcomes	Level 4	(Satellite)	Patient safety program includes review of all medication adverse events and system improvement.
			Quality indicators:
			Assessment of toxicities and documentation of adverse reaction.
	Level 3	(Affiliate)	Same as level 4.
	Level 2	(icp)	Same as level 3.
	Level 1	(icp)	Same as level 2.
Organization outcomes	Level 4	(Satellite)	Multidisciplinary cancer conference participation encouraged.
			Quality indicators:
			Track volume of patients treated.
			Other indicators such as monitoring adherence to guidelines.
	Level 3	(Affiliate)	Level 4, plus:
			Multidisciplinary cancer conference participation required.
	Level 2	(icp)	Same as level 3.
	Level 1	(icp)	Same as level 2.
System outcomes	Level 4	(Satellite)	Quality indicators:
			Percentage of patients treated close to home.
	Level 3	(Affiliate)	Level 4, plus:
			Monitoring systemic treatment wait times.
	Level 2	(icp)	Same as level 3.
	Level 1	(icp)	Same as level 2.

icp = integrated cancer program.

**TABLE VII:** Facility requirements

All levels of care	The necessary infrastructure is in place to provide the service level.		
Clinic space	Level 4	(Satellite)	Dedicated systemic treatment area adequate for volume of treatment visits.
			Adequate space to provide clinical trials if applicable.
	Level 3	(Affiliate)	Same as level 4.
	Level 2	(icp)	Same as level 3.
	Level 1	(icp)	Level 2, plus:
			Dedicated clinical trials infrastructure on site.
Clinic equipment	Level 4	(Satellite)	Computer, facsimile, and telephone accessibility.
			Computer software available to provide computerized physician order entry.
	Level 3	(Affiliate)	Same as level 4
	Level 2	(icp)	Same as level 3.
	Level 1	(icp)	Same as level 2.
Systemic treatment and facility safety equipment	Level 4	(Satellite)	Oxygen.
			Biological safety cabinet (class 2) and externally vented.
			Appropriate tubing, Luer-lock syringes.
			Intravenous equipment for parenteral therapy.
			Intravenous equipment for ambulatory or inpatient infusional therapy (pumps).
			Personal protective equipment for staff who are handling systemic treatment or waste.
			Spill kits and supplies for decontamination.
			Emergency resuscitation equipment (for example, crash cart, other emergency supplies, drugs, oxygen, and suction) in case of cardiorespiratory arrest or anaphylaxis.
			Supportive drugs for treatment of extravasation.
			Designated clinical trial storage if doing clinic trials.
	Level 3	(Affiliate)	Same as level 4.
	Level 2	(icp)	Same as level 3.
	Level 1	(icp)	Same as level 2.
Institutional facilities	Level 4	(Satellite)	Emergency department.
			Pharmacy for secure storage and preparation of systemic treatment drugs.
			Access to inpatient beds for oncology patients.
			Access to local specialized diagnostic imaging (computed tomography, ultrasound, nuclear medicine), laboratory tests, and pathology for the monitoring of systemic treatment.
			Access to intensive care unit.
			Access to facility for insertion of central venous catheters or Port-A-Cath access devices.
			Potential for videoconferencing, remote Web-based teaching, and patient management as part of multidisciplinary cancer conference.
	Level 3	(Affiliate)	Same as level 4, plus:
			Intensive care unit and specialized diagnostic imaging on site.
	Level 2	(icp)	Level 3, plus:
			Radiation therapy services on site.
			Pathology services on site.
			Magnetic resonance imaging on site.
			Specialized diagnostic imaging on site.
	Level 1	(icp)	Same as level 2.

icp = integrated cancer program.

**TABLE VIII:** Administrative and organizational responsibilities

All levels of care	Should measure common provincial indicators.		
	May also measure regional indicators as defined by the regional cancer program.		
Data reporting requirements	Level 4	(Satellite)	Outcome indicators that are specific; measurable; attainable, achievable, action-oriented; relevant; time-framed (SMART 16).
			Decision-support resources to collate and analyze quality indicators.
	Level 3	(Affiliate)	Same as level 4.
	Level 2	(icp)	Same as level 3, plus:
			Compliant with Cancer Care Ontario data book.
	Level 1	(icp)	Same as level 2.
Leadership	Level 4	(Satellite)	Physician and administrative leads identified, with defined roles to manage strategic and operational issues through regional forums.
			Formal linkage to a regional systemic treatment program.
			Nursing and pharmacy administrative leads identified, with defined roles to manage strategic and operational issues through the regional systemic treatment program.
	Level 3	(Affiliate)	Level 4, plus:
			May have formalized linkages with a satellite.
	Level 2	(icp)	Level 3, plus:
			Regional vice president and regional systemic treatment leads.
	Level 1	(icp)	Same as level 2.
Logistics support	Level 4	(Satellite)	Clerical staff and clinic facilities to support patient scheduling, health records management, and clinic management including clinic and administrative supplies for systemic treatment suites and ambulatory clinic visits.
	Level 3	(Affiliate)	Same as level 4.
	Level 2	(icp)	Same as level 3.
	Level 1	(icp)	Same as level 2.
Information systems	Level 4	(Satellite)	Information system hardware and support to maintain a secure electronic systemic treatment order entry program and other electronic systems as indicated (for example, electronic patient record).
	Level 3	(Affiliate)	Same as level 4.
	Level 2	(icp)	Same as level 3.
	Level 1	(icp)	Same as level 2.

icp = integrated cancer program.

## Definitions

### Advanced Oncology Nurse

The advanced oncology nurse is prepared at the Master’s level (mscn or equivalent). Additional certification as an acute care nurse practitioner (or other levels) may be acquired either within the graduate program or as a postgraduate course and certification. The domains of the advanced oncology nurse include

- advanced clinical practice,
- education,
- research,
- scholarly/professional leadership, and
- organizational leadership^15^.

### Certification in Systemic Treatment Administration (Certified in Chemotherapy)

No registered nurse in Ontario should administer intravenous systemic treatment until and unless that nurse has received additional education and has demonstrated competency in the delivery of these cytotoxic agents. This requirement is specific to the delivery of chemotherapy and is not to be confused with the national examination process for certification as an oncology nurse through the Canadian Nurses Association.

### Complexity

Complexity is determined by the preparation and administration requirements for systemic treatment, risk of immediate grade 3 or 4 toxicities, medical condition of the patient, or use of investigational agents or new agents just approved for which little long-term toxicity data are available.

### Institutional Facilities

Hospitals, clinics, or offices as outlined in the facility requirements element.

### Integrated Cancer Program (icp)

A multidisciplinary in-and-out patient cancer program including medical, radiation, and surgical oncology. The icp will also provide research, education, and organizational leadership for the regional cancer program.

### Oncologist

A physician with subspecialty training in the administration of systemic treatment, recognized by the Royal College of Physicians and Surgeons of Canada, including medical oncologists, hematologists, and gynecologic oncologists.

### Quality Indicator

A specific, measurable, attainable, relevant, time-framed outcome from the patient, organizational, or system perspective to assess performance^16^.

### Regional Systemic Treatment Program (rstp)

An agreed-upon relationship between satellites, affiliates, and icps.

### Specialized Oncology Nurse

A nurse who has a combination of expanded education focused on cancer care and experience such as 2 years in a setting in which the primary focus is cancer care delivery. The specialized oncology nurse might acquire specialty education in a variety of ways—for example, enrolment in an undergraduate nursing program, completion of an oncology certificate program, distance specialty education (such as that offered in adult and pediatric oncology nursing), or registration in and completion of the certification exam offered by the Canadian Nurses Association and attainment of the distinction Certified in Oncology Nursing (Canada).

The specialized oncology nurse works in a specialized inpatient setting such as an oncology unit or bone-marrow transplant unit, an ambulatory setting focused on the delivery of cancer care, a screening program, or a supportive care setting or community setting offering palliative care. The individual’s enhanced specialty knowledge and skill can be utilized in many environments to manage symptoms and side effects of treatment, to counsel patients in coping strategies, to teach self-care behaviours, and to monitor responses to treatment and nursing interventions^15^.

### Systemic Treatment

Any oral or parenteral, hormonal, biologic, chemotherapeutic, or radiopharmaceutic anticancer agent.
